# Differential expression of sirtuin 2 and adipocyte maturation restriction: an adaptation process during hypoxia in fish

**DOI:** 10.1242/bio.027334

**Published:** 2017-08-14

**Authors:** Padmini Ekambaram, Parimala Parasuraman

**Affiliations:** P.G. Department of Biochemistry, Bharathi Women's College, Affiliated to University of Madras, Tamil Nadu, Chennai-600 108, India

**Keywords:** Adipokines, Adipogenic transcription factors, Anti-adipogenic markers, Field hypoxia, *In vitro* hypoxia

## Abstract

Sirtuins have received widespread attention due to their diverse physiological role in metabolism. Among sirtuins, SIRT2 is more abundant in adipocytes and exerts effects on adipocyte differentiation, a process which involves conversion of preadipocytes to mature adipocytes orchestrated by adipokines and adipogenic transcription factors. Grey mullet (*Mugil cephalus*) was chosen as a study organism due to its excellent service as a biomonitor. Adipocytes isolated from natural field conditions were termed as field-hypoxic (Ennore) and -normoxic (Kovalam) based on dissolved oxygen (DO) level in the estuary. A previous study portrayed the hypoxic instance of Ennore estuary (low DO) and grey mullet [HIF1α in adipocytes, brain endothelial cell (EC) and hepatocytes] inhabiting this estuary ( Padmini et al., 2016a, b; Padmini and Tharani, 2015). In this context, fish adipocytes of both conditions were subjected to *in vitro* hypoxia for 1 h (in the pre/trigassed incubator with the supply of 1% O_2_; 94% N_2_; 5% CO_2_) and were analysed for the expression of adipokines, adipogenic transcription factors and anti-adipogenic markers in fish adipocytes. Elevation of asymmetric dimethylarginine (ADMA), TNFα and leptin along with decreased adiponectin, adipogenic transcription factors and altering sirtuins were observed in test adipocytes and in control adipocytes on *in vitro* hypoxia. This suggests that adipocytes may follow internal caloric restriction as portrayed from cytomorphological/ultrastructural analysis, limiting adipocyte maturation process, one of the adaptive mechanisms triggered by adipocyte of fish surviving in Ennore estuary. Prolonged exposure to hypoxia (test on *in vitro* hypoxia for 1 h) showed a drastic alteration in these components leading to both structural and biological fluctuation when compared to limited hypoxic condition (field-hypoxic and control on *in vitro* hypoxia). Our study concludes that hypoxia may serve as the chief molecular cue in eliciting adipocyte maturation restriction though metabolic reprogramming and it also shows the significance of adipocyte maturation restriction in imparting survival mechanism.

## Abstract (precis)

**Summary:** Adipocyte maturation restriction is tightly regulated by SIRT2 activation which downregulates preadipocytes from the maturation process as adaptation strategy in fish surviving in the polluted (hypoxic) environment.

## INTRODUCTION

Aquatic organisms tolerate stress by different adaptive mechanisms, either by caloric restriction or by regulating metabolic flux to facilitate the energy homeostasis which in turn raises the maximum chances of survival (Leal et al., 2011). In the present study, grey mullets (*Mugil cephalus*) were chosen as the study organism as they revealed a significant observation from the cohort studies with comparative analysis pursued over a decade. Adipocytes isolated from grey mullet were analysed due to their dynamic role in regulating energy homeostasis (under conditions of energy surfeit and energy deficit) and endocrine functions, and also serve as a storage organ for many toxic chemicals (due to their lipophilicity) thereby trapping many xenobiotics and preventing their distribution to other organs (Marisa et al., 2013; Lee et al., 2017). Though adipose tissue requires low oxygen for its physiological function (Elia, 1992), hypoxia beyond the threshold limit may result in adipose dysfunction (Padmini et al., 2016a). Coherent to this, our previous study observed that adipocytes isolated from Ennore estuarine fish challenge severe hypoxia, which may be attributed to the prevalence of hypoxia in Ennore estuary as depicted from the low dissolved oxygen (DO) level. Furthermore, induction of lipolysis and suppression of lipogenesis along with decreased mature adipocytes depicts that adipocytes encounter the maturation restriction process to maintain cell homeostasis under hypoxia (Padmini et al., 2016a; Padmini and Parimala, 2017). The effect of hypoxia in grey mullet's brain endothelial cells and hepatocytes was also demonstrated, which clearly portrays the effect of xenobiotics in persuading hypoxia and its mediated effect in their cytological system (Padmini et al., 2016b; Padmini and Tharani, 2015). These studies suggest that hypoxia is the conclusive effect of xenobiotics accumulation in the aquatic system and organism. Hypoxic quality of Ennore estuary may be attributed to the vast environmental pollutants diffusion which includes domestic wastes (detergents), agricultural wastes (fertilisers, pesticides), and industrial wastes (thermal effluents, heavy metals, organic pollutants) (Natesan, 2013; Shanthi and Ramanibai, 2011; Chitrarasu et al., 2013; Padmini and Usha Rani, 2009). In this context, *in vitro* hypoxic model system (detailed information given in Materials and methods) was adopted to understand the hypoxic (field) impression on adipogenesis in particular adipocyte maturation by assessing anti-adipogenic factors [SIRT2, asymmetric dimethylarginine (ADMA)], adipokines (Leptin, adiponectin, TNFα), and adipogenic transcription factors (PPAR-γ and CEBP-β).

Sirtuins act as the major sensor of energetic status. They are a highly conserved family of proteins that mediate cellular physiology and energy demands in response to metabolic inputs (Priyanka et al., 2015). Unlike other protein deacetylases, sirtuins require nicotinamide adenine dinucleotide (NAD) as a cofactor in the deacetylation reaction (Imai et al., 2000). SIRT2 was found to be the most abundant sirtuin in adipocytes and plays a critical role in regulating adipocyte differentiation, thereby maintaining adipogenesis (Jing et al., 2007). Hence, monitoring SIRT2 and its link with adipocyte metabolism via assessing triglycerides (TG) and G3PDH may provide novel insight into the fish adipocyte maturation under hypoxic stress. The abundance of SIRT2 [NAD-dependent deacetylase (NDAC)] increases dramatically during mitosis and phosphorylated at the G2/M transition of the cell cycle, indicating that it plays a central role in mitosis (Dryden et al., 2003). Since exit from the cell cycle is the prerequisite for the conversion of preadipocytes to mature adipocytes (Rosen and Spiegelman, 2000), ascertaining SIRT2 expression may act as a novel tool in defining the adipocyte maturation process.

Adipocyte acts as a major signalling unit, secreting a multiplicity of protein factors, the adipokines (Trayhurn, 2013). Netzler et al. (2015) reported that hypoxia can provoke oxidative stress in human and animal adipocytes and alter the production of adipokines. Oxidant stress is an important mediator of adipocyte dysfunction. These adipokines include leptin, adiponectin, resistin, tumour-necrosis factor α, interleukin 6, chemokine (C–C motif) ligand 2, interleukin 10, transforming growth factor-β and HO-1 (Pereira and Alvarez-Leite, 2014). Adipokines function as classic circulating hormones to interact with other organs, including brain, liver, muscle, the immune system and adipose tissue itself. Alteration of adipokines leads to the development of either metabolic adaptations or disturbances. Extensive studies have been established and the level of adipokines in mammals with relation to insulin resistance, obesity and glucose metabolism during metabolic and environmental stress has been analysed (Shane and Robert, 2014). To the best of our knowledge, the assessment of adipokines has been demonstrated for the first time in adipocytes of grey mullet, and it may be a valuable contribution to understand the function of adipokines in fish during pollutants mediated stress and its role in the regulation of lipid metabolism in adipocytes.

Many peptides (adipokines) are involved in the interplay among appetite, metabolic rate and energy stores of which leptin is considered to be the superior (Copeland et al., 2011). It is a 16 kDa non-glycosylated peptide hormone, consisting of 167 amino acids, primarily secreted by differentiated and mature adipocytes. Its expression is altered by overfeeding, fasting, insulin, glucocorticoids, endotoxin, cytokines, testosterone and thyroid hormone (Yang and Lili, 2007). Leptin binds to its receptors (LEPR) and activates the Janus kinase (JAK)/signal transducer and activator of transcription (STAT) signalling pathway (Copeland et al., 2011). Its production is positively related to the expansion of adipose tissue and total body fat.

Adiponectin (ACRP30, adipoQ, apM1, or GBP28) is another important adipokine induced during adipocyte differentiation. It is a 30-kDa protein, synthesised and secreted by adipocytes. It acts as the prime regulatory protein in various physiological pathways to control lipid and carbohydrate metabolism. In plasma, adiponectin circulates as either a trimer, a hexamer [called the low molecular weight (LMW) form], or as multimeric forms of 12 to 18 subunits [called the high molecular weight (HMW) form] (Kadowaki et al., 2006). Hao and Henry (2005), report that the HMW form may undergo cleavage to form smaller units, which functions as the active form to transduce adiponectin's signal to cells. Activated adiponectin acts mainly via two receptors, AdipoR1 and AdipoR2. Transduction of the adiponectin signal by AdipoR1 and AdipoR2 involves the activation of AMPK, peroxisome proliferator activated receptor (PPAR; α and γ) and presumably other signalling molecules (Berg and Scherer, 2005) to induce biological functions.

Another significant adipokine is TNFα, is a 26-kDa transmembrane protein released into the circulation as a 17-kDa soluble protein after extracellular cleavage by a metalloproteinase (Gearing et al., 1994). TNFα exhibits its functions through its binding to two main receptors, type 1 and 2, which are expressed in many cells including adipocytes (Hotamisligil et al., 1997). TNFα has multiple effects on lipid metabolism, via paracrine effects on adipocytes and liver. In adipose tissue, TNFα promotes lipolysis (Green et al., 1994), leading to elevation of plasma free fatty acid (FFA) levels. Additionally, TNFα causes reductions in the expression of genes involved in adipogenesis and lipogenesis in adipocytes, likely through NFκβ-mediated transcription. TNFα significantly alters the expression of adiponectin, IL-1 and IL-6, thereby affecting the lipid metabolism (Ruan et al., 2002).

Adipogenesis is a multi-step process involving a cascade of transcription factors including CCAAT/enhancer-binding protein (C/EBP) gene family and PPAR-γ (Lefterova and Lazar, 2009). During adipocyte differentiation, remarkable changes occur in cell morphology and cytoskeletal components. Both PPAR-γ and C/EBP-α appear to act co-operatively during adipocyte differentiation by activating the expression of one another and regulating the expressions of other adipocyte-specific genes (Mandrup and Lane, 1997). They are the pivotal coordinators of the adipocyte differentiation process, by inducing the cell to exit from the cell cycle and triggering the expression of adipocyte-specific genes, resulting in increased delivery of energy to the cells resulting in mature fat cells (Rosen and Spiegelman, 2000). Prior to PPAR-γ, C/EBP family members C/EBP-β and C/EBP-δ are expressed and involved in adipogenic induction at an earlier stage than PPAR-γ by inducing the expression and/or activity of PPAR-γ (Guo et al., 2015). In a previous study, we noted the alteration in the level of fatty acid (FA) and glycerol under field and *in vitro* hypoxic condition which partially depicts the hypoxia-mediated disruption in adipogenesis and adipocyte maturation (Padmini et al., 2016). Hence adipogenic process was evaluated through their transcription factors in the present study to further understand how hypoxia mediates the effect on adipogenesis.

Existence of adipocyte hypoxia has been linked to dysregulation of adipose tissue blood flow (Netzler et al., 2015). Importantly, all metabolic processes in adipose tissue depend on blood supply (Sotornik et al., 2012). May et al. (2014) demonstrated that ADMA in adipose tissue plays a role in NO-dependent regulation of adipose tissue blood flow and metabolism. Zhou et al. (2009) reported that the level of ADMA is indirectly proportional to the adipogenesis revealing that it acts as a potential marker of adipocytes status. Fundamentally, ADMA is an endogenous inhibitor of nitric oxide synthase (NOS) which is formed by the addition of methyl groups to the guanidino residues of arginine, catalysed by the protein arginine methyltransferase (PRMT) family of enzymes (Pahlich et al., 2006). Arginines can either be mono-methylated (L-NMMA) or di-methylated on either the same guanidino group (ADMA) or one on both guandino groups (symmetric dimethylarginine, SDMA) (Bedford and Clarke, 2009). However, generated ADMA is continuously metabolized by the enzyme dimethylarginine dimethylaminohydrolase (DDAH) during normal and acute stress conditions. During chronic stress conditions, increase in ADMA concentrations may occur due to the inhibition of DDAH. Hence, quantification of ADMA will directly reflect the adipocyte environment and its mediated effect on metabolic status.

Deciphering adipocyte cell biology is an important component of understanding how the adipocyte contributes to the metabolic regulation associated with aberrant stress induced by pollutants. Our earlier study on fish adipocytes establishes that hypoxia has pervasive effects on fish adipocytes substantiated by subjecting adipocytes to *in vitro* hypoxia for 1 h. It depicted the coordinated expression of HIF1α and HSP70 in rendering cytoprotective mechanism by suppressing ASK1 and p-JNK1/2 during hypoxia-induced stress conditions (Padmini et al., 2016). It also revealed the decrease in the mature adipocytes isolated from fish of Ennore estuary; depicting that an intricate phenomenon should be involved in influencing adipogenesis during pollutants mediated hypoxic stress. We therefore investigated key adipokines and the adipogenic markers along with analysing SIRT2 in adipocytes isolated from the field hypoxic condition to get more insight into the adipocyte maturation process under hypoxic stress. These adipocytes were further subjected to hypoxia in the laboratory to investigate whether extended hypoxia will alter the field hypoxic effect.

## RESULTS

### Cell viability

**Fig. 1.**
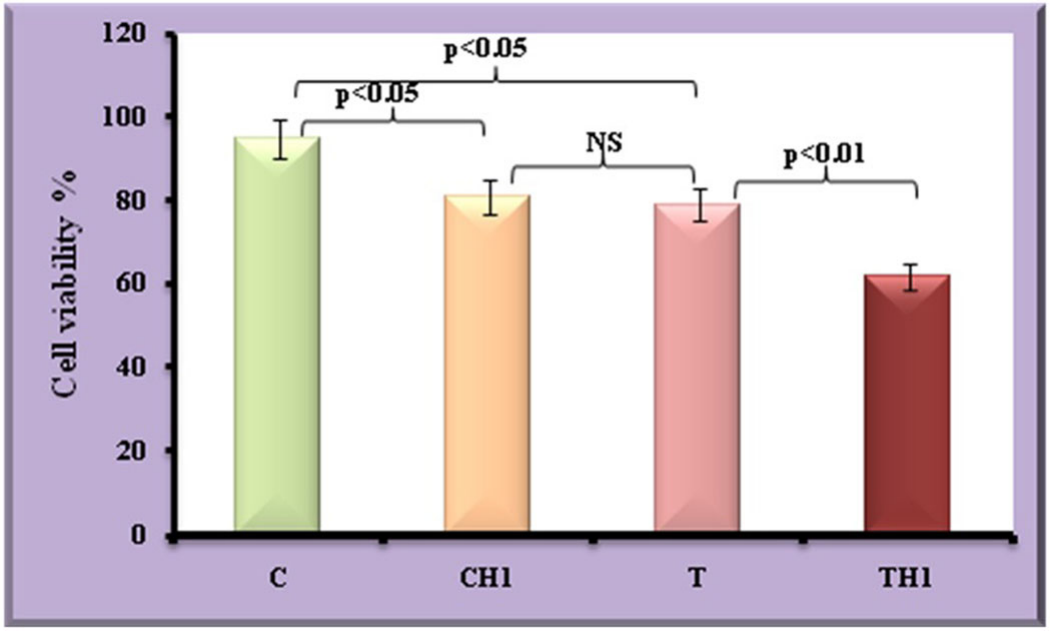
**Viability of adipocytes under field and *in vitro* hypoxia.** Values are expressed as means±s.d. (*n*=20 fish per site, Student's *t*-test). C, control adipocytes; T, test adipocytes; CH, control adipocytes under hypoxia; TH, test adipocytes under hypoxia.

Fig. 1 depicts the cell viability. Decrease in the cell viability (17%; *P*<0.05) observed in test adipocytes when compared to control adipocytes.

### Histochemistry

**Fig. 2.**
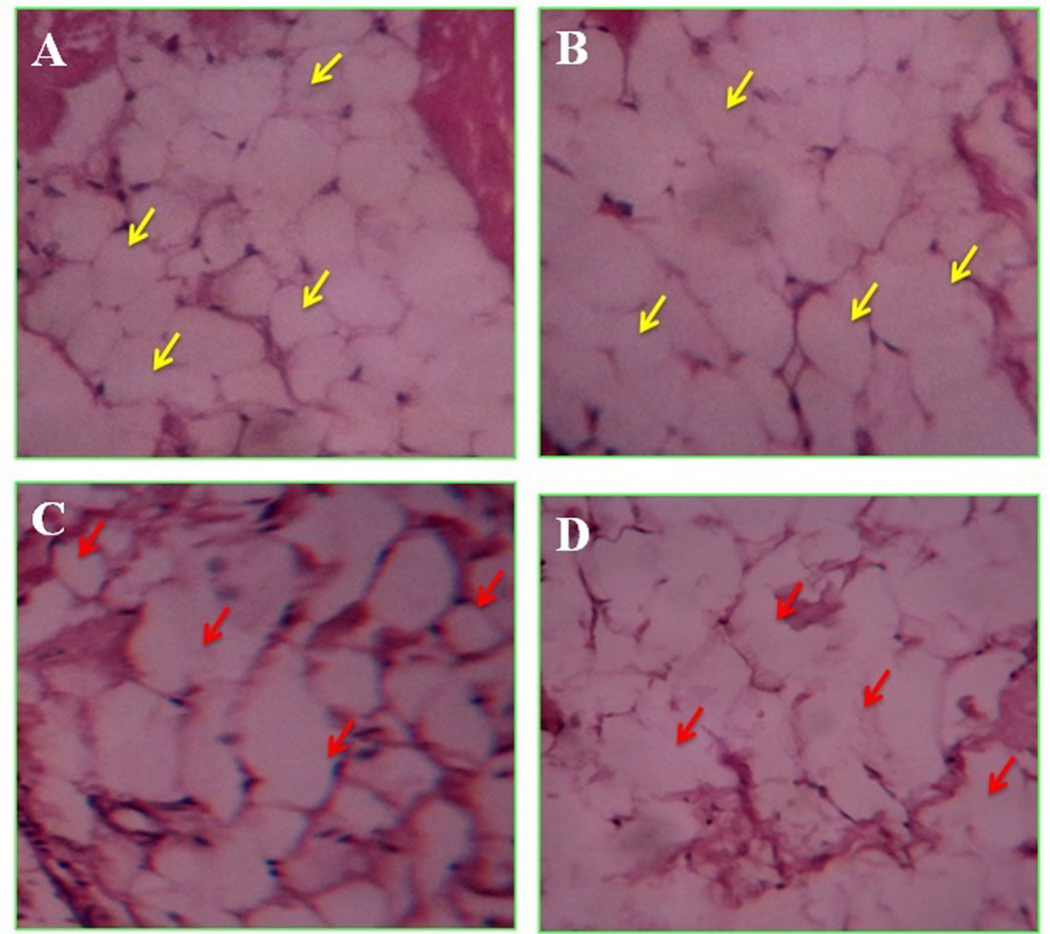
**Cytopathological evaluations of haematoxylin and eosin stained adipocytes of grey mullet from Kovalam and Ennore estuaries.** (A,B) Kovalam and (C,D) Ennore estuaries, 40× magnification. Yellow arrows indicate control adipocytes with regular shape, firm architecture and membrane integrity. Red arrows indicate test adipocytes with irregular shape, distinguishing structural characteristics and disturbed membrane.

Fig. 2 depicts the histochemical evaluation of adipocytes stained with eosin-hematoxylin. Numerous fat cell clusters with firm structural architecture is observed in control adipocytes. Morphological differentiation characterised with disrupted membrane integrity in adipocytes under field and *in vitro* hypoxic conditions depicts the metabolic competence, i.e. lipolytic process.

### Transmission electron microscopy

**Fig. 3.**
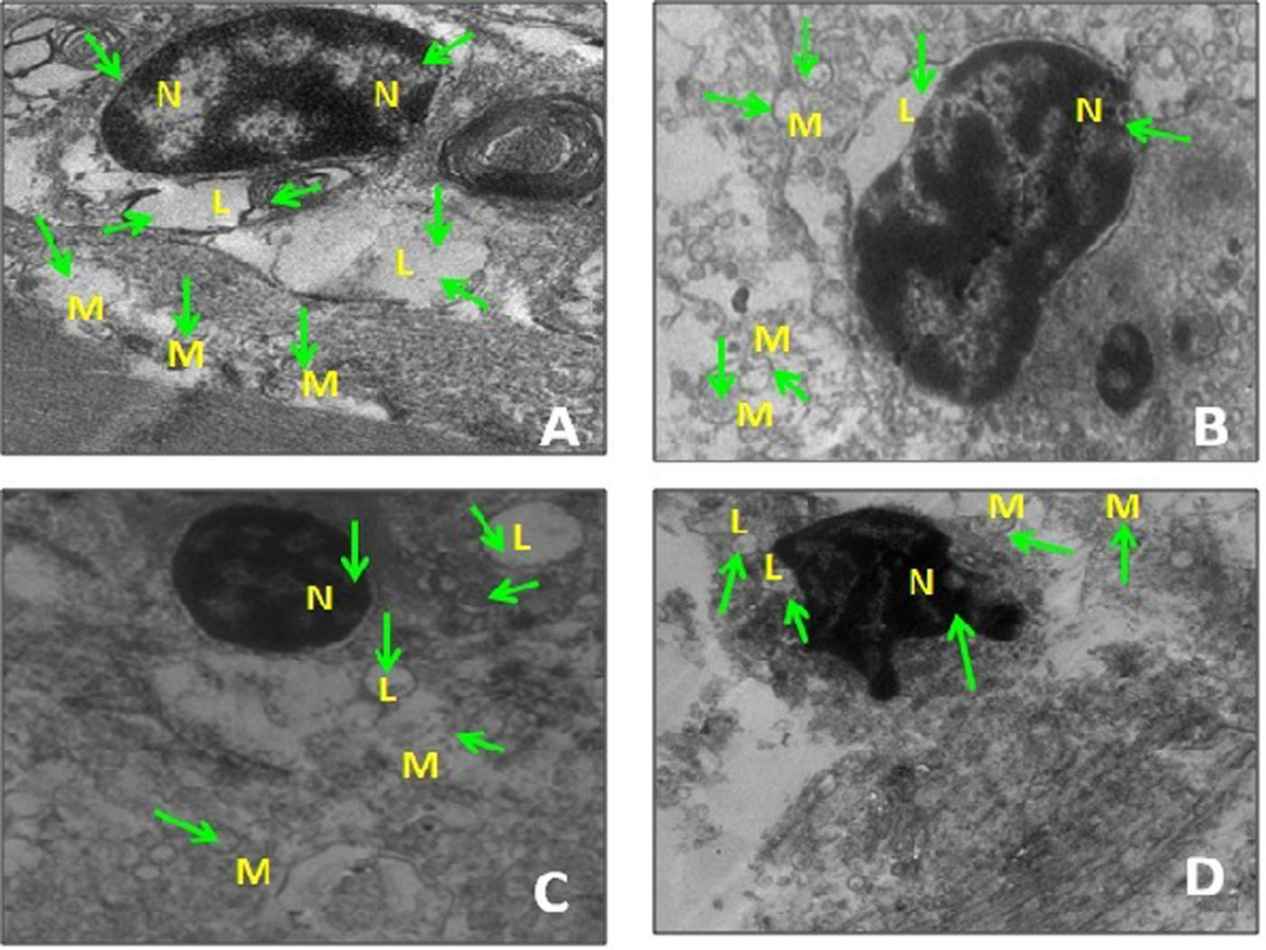
**Transmission electron micrograph of adipocytes of *Mugil cephalus* under field and *in vitro* hypoxia.** Original magnification: ×9900. (A) Control adipocytes. (B) Test adipocytes (field hypoxic). (C) Control adipocytes on *in vitro* hypoxia. (D) Test adipocytes on *in vitro* hypoxia. N, nucleus; L, lipid droplet; M, mitochondria. Green arrows indicate subcellular organelles.

Fig. 3 demonstrates the transmission electron micrographs of adipocytes of field hypoxic and *in vitro* hypoxic conditions. It highlights the presence of large lipid droplets in control adipocytes (Fig. 2A), small lipid droplets in test adipocytes and control adipocytes on *in vitro* hypoxia (Fig. 2B,C), and further smaller lipid droplets in test adipocytes on *in vitro* hypoxia (Fig. 2D) indicated by the arrows. It also portrays the presence of well-preserved organelles such as nucleus (N), mitochondria (M) and endoplasmic reticulum (ER) in control adipocytes; whereas in test adipocytes we observed control and test adipocytes on *in vitro* hypoxia altered structural disturbances of cell organelles such as nucleus (N), mitochondria (M) and endoplasmic reticulum (ER).

### Adipocyte maturation reflecting markers

**Fig. 4.**
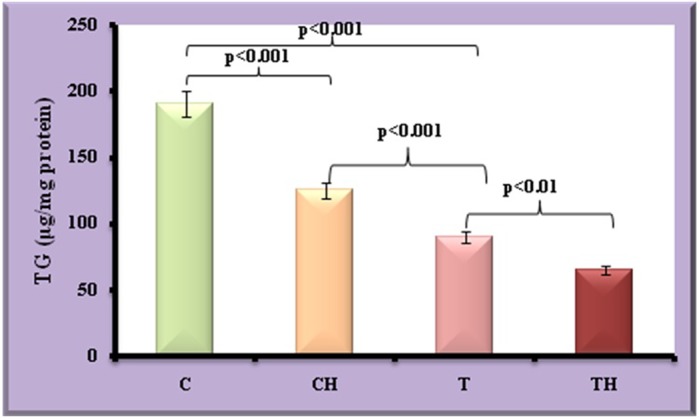
**Level of TG in fish adipocytes under field and *in vitro* hypoxic condition.** Values are expressed as means±s.d. (*n*=20 fish per site, Student's *t*-test). C, control adipocytes; T, test adipocytes; CH, control adipocytes under hypoxia; TH, test adipocytes under hypoxia.

**Fig. 5.**
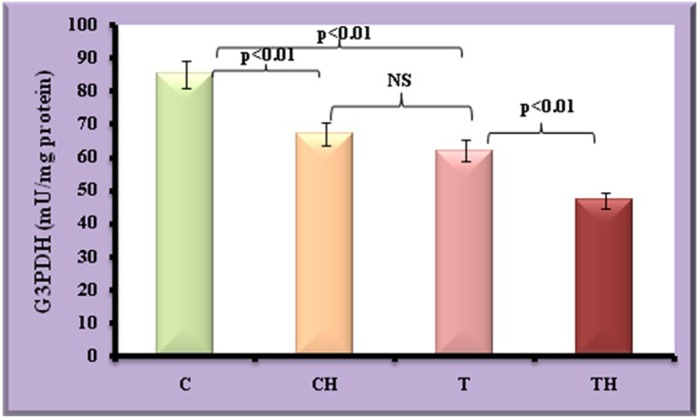
**Level of G3PDH in fish adipocytes under field and *in vitro* hypoxic condition.** Values are expressed as means±s.d. (*n*=20 fish per site, Student's *t*-test). C, control adipocytes; T, test adipocytes; CH, control adipocytes under hypoxia; TH, test adipocytes under hypoxia.

Figs 4 and 5 depict the level of TG and G3PDH which directly reflect the status of adipocyte maturation. Decreased TG and G3PDH was observed in test adipocytes (53%, *P*<0.001 and 27%, *P*<0.01) and control adipocytes on *in vitro* hypoxia (34%, *P*<0.001 and 21%, *P*<0.01) compared to control adipocytes. On *in vitro* hypoxia further decrease in the TG and G3PDH were observed in test adipocytes (28%, *P*<0.01 and 30%, *P*<0.01).

### Adipokines

**Fig. 6.**
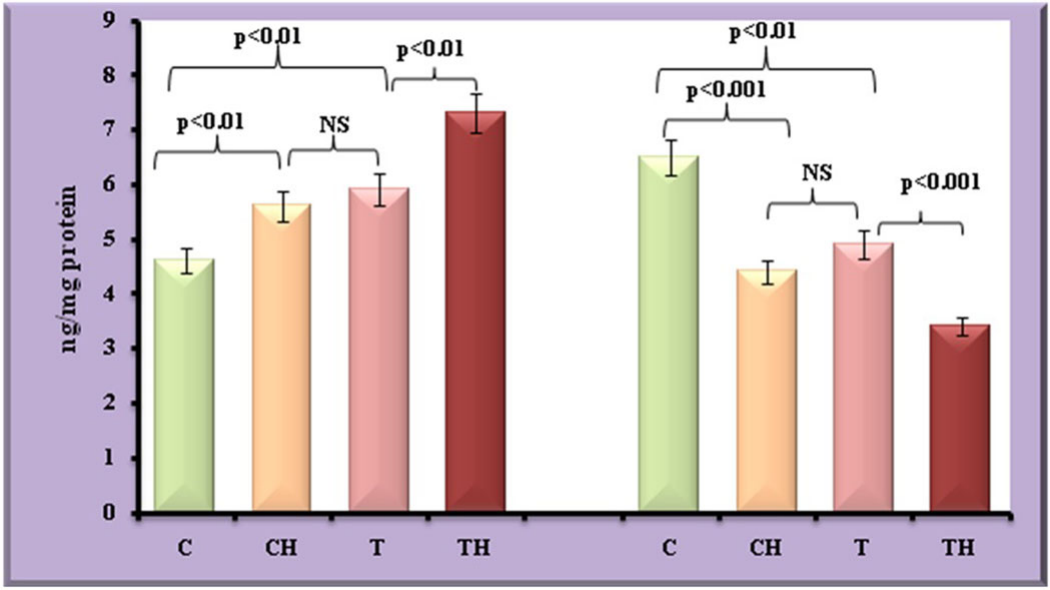
**Level of leptin and adiponectin in fish adipocytes under field and *in vitro* hypoxic condition.** Values are expressed as means±s.d. (*n*=20 fish per site, Student's *t*-test). C, control adipocytes; T, test adipocytes; CH, control adipocytes under hypoxia; TH, test adipocytes under hypoxia.

**Fig. 7.**
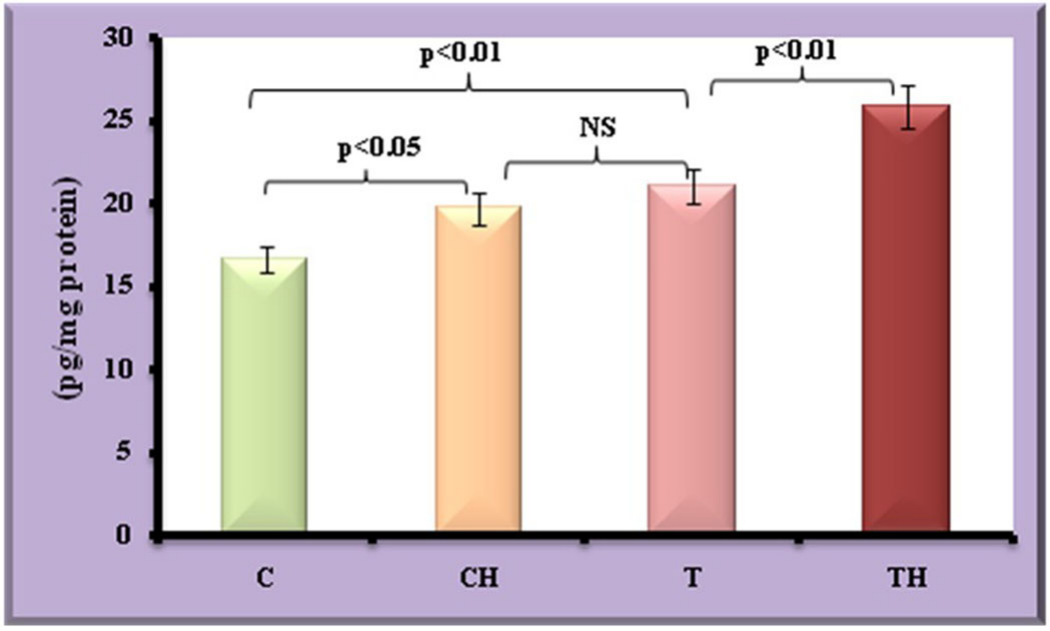
**Level of TNFα in fish adipocytes under field and *in vitro* hypoxic condition.** Values are expressed as means±s.d. (*n*=20 fish per site, Student's *t*-test). C, control adipocytes; T, test adipocytes; CH, control adipocytes under hypoxia; TH, test adipocytes under hypoxia.

Figs 6 and 7 depict the differential expression of adipokines such as leptin, adiponectin and TNFα under field and *in vitro* hypoxia.

### Leptin

Increase in the expression of leptin (28%, *P*<0.01) was observed in test adipocytes compared to control adipocytes. On *in vitro* hypoxia, increase in the expression of leptin by 22% (*P*<0.01) in control adipocytes and further increase in leptin by 24% (*P*<0.01) was observed in test adipocytes.

### Adiponectin

Decrease in the expression of adiponectin (25%, *P*<0.01) was observed in test adipocytes compared to control adipocytes. On *in vitro* hypoxia, decrease in the expression of adiponectin by 32% (*P*<0.001) in control adipocytes and further increase in adiponectin by 31% (*P*<0.001) was observed in test adipocytes.

### TNFα

Increase in the expression of TNFα in test adipocytes (27%, *P*<0.01) and control adipocytes on *in vitro* hypoxia (19%, *P*<0.05) were observed. Further increase in TNFα was observed in test adipocytes on *in vitro* hypoxia (23%, *P*<0.01).

### Adipogenic transcription factors

**Fig. 8.**
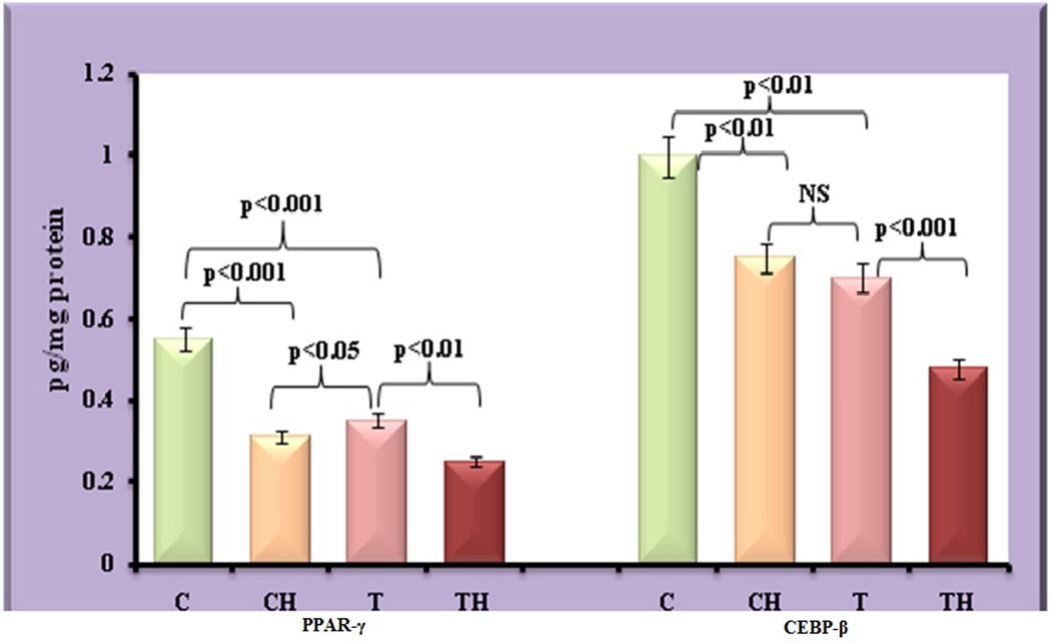
**Level of PPAR-γ and C/EBP-β in fish adipocytes under field and *in vitro* hypoxic condition.** Values are expressed as means±s.d. (*n*=20 fish per site, Student's *t*-test). C, control adipocytes; T, test adipocytes; CH, control adipocytes under hypoxia; TH, test adipocytes under hypoxia.

Fig. 8 depicts the adipogenic transcription factors.

### PPAR-γ and C/EBP-β

Decrease in the expression of PPAR-γ and C/EBP-β in test adipocytes (36%, *P*<0.001 and 30%, *P*<0.01) and control adipocytes on *in vitro* hypoxia (44%, *P*<0.001 and 25%, *P*<0.01) were observed. However, on *in vitro* hypoxia further decrease in PPAR-γ and C/EBP-β was observed in test adipocytes (29%, *P*<0.01 and 31%, *P*<0.001).

### Anti-adipogenic markers

**Fig. 9.**
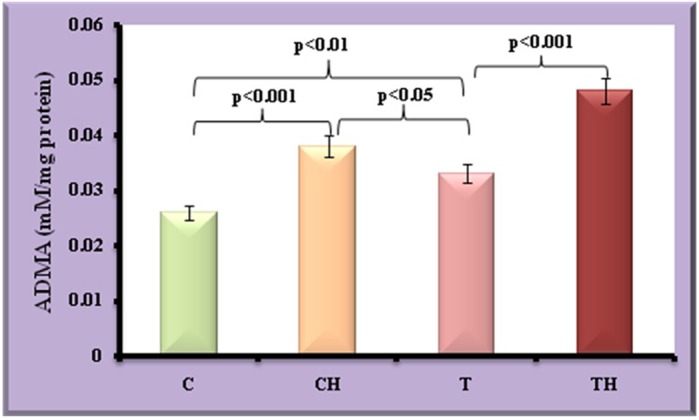
**ADMA in fish adipocytes under field and *in vitro* hypoxic condition.** Values are expressed as means±s.d. (*n*=20 fish per site, Student's *t*-test). C, control adipocytes; T, test adipocytes; CH, control adipocytes under hypoxia; TH, test adipocytes under hypoxia.

**Fig. 10.**
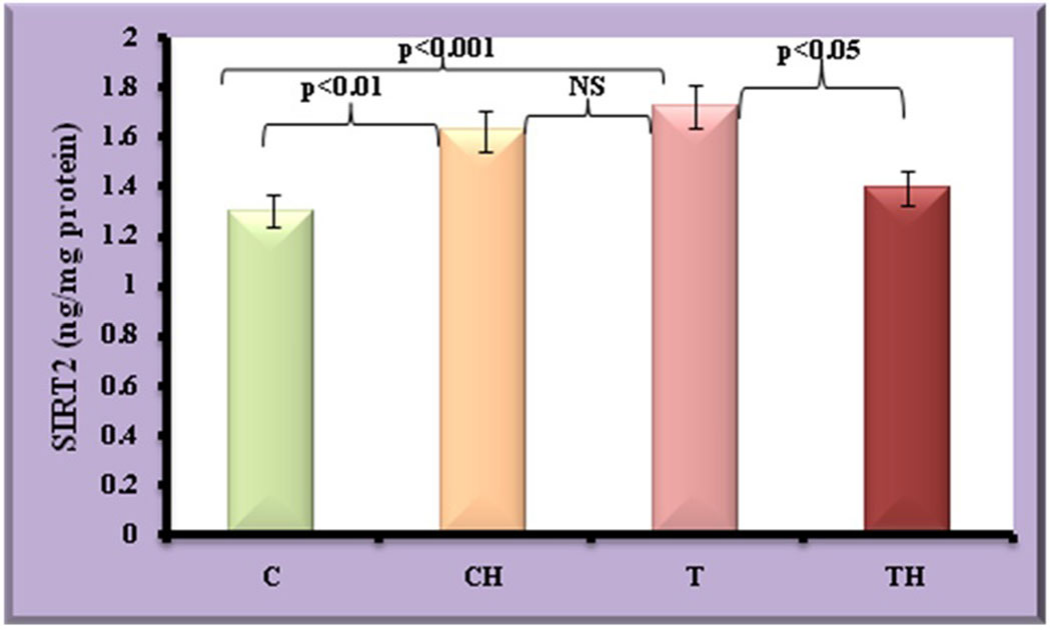
**Level of SIRT2 in fish adipocytes under field and *in vitro* hypoxic condition.** Values are expressed as means±s.d. (*n*=20 fish per site, Student's *t*-test). C, control adipocytes; T, test adipocytes; CH, control adipocytes under hypoxia; TH, test adipocytes under hypoxia.

Anti-adipogenic markers such as ADMA and SIRT2 were given in Figs 9 and 10. Increase in ADMA and SIRT2 were observed in test adipocytes (27%, *P*<0.01 and 32%, *P*<0.001) when compared to control adipocytes. On *in vitro* hypoxia, further increase in ADMA (46%, *P*<0.001) and SIRT2 (25%, *P*<0.01) were observed in control fish adipocytes. Whereas in test adipocytes subjected to *in vitro* hypoxia, further elevation in ADMA (45%, *P*<0.001) and decline in SIRT2 (19%, *P*<0.05) were observed.

## DISCUSSION

In this study we have tried to identify the role of SIRT2 in the fish adipocyte maturation process during hypoxia. Generally the functional changes in adipocyte in response to low dissolved oxygen is elucidated from the rates of lipolysis and lipogenesis. Decreased levels of TG and G3PDH during hypoxia exhibit the existence of lipolytic process in these cells. Eventually the adipocyte maturation is altered during hypoxia which is represented by the histochemical evaluation and EM pictures of adipocytes. Histochemical evaluation represents numerous fat cell clusters with firm structural architecture in control adipocytes; whereas morphological differentiation characterised with disrupted membrane integrity is observed under field and *in vitro* hypoxic conditions depicting the metabolic competence. Alteration in the size of lipid droplets and the cell organelles under hypoxic conditions also supports the suppression of adipocyte maturation process.

Further the expression of adipokines also evidences the effect of hypoxia on adipocyte maturation. It is demonstrated from the expression of leptin which is inversely correlated to the expression of adiponectin during hypoxia (Hosogai et al., 2007; Ye et al., 2007; Trayhurn, 2013). Low dissolved oxygen instigates the adipocytes into leptin secreting cells prior to their differentiation into mature adipocytes (Wang et al., 2008). Similarly, increased expression of leptin observed in fish adipocytes under hypoxic conditions depicts the intriguing effect of hypoxia in adipocyte maturation. Moreover, recent studies have indicated that HIF-1 binds to the leptin gene promoter during hypoxia and stimulates its transcription (Wang et al., 2008; Grosfeld et al., 2002). This may be owing to the binding affinity of HIF-1α to leptin gene promoter to stimulate its transcription during hypoxia. Cabrero et al. (2005) reported that enhanced leptin downregulates the expression of PPAR-γ, key adipogenic transcription factor. Consistent with this, we have noted an increase in leptin, and a decrease in adipogenic transcription factors. The oxidative stress experience by adipocytes reduces the levels of beneficial adiponectins (Netzler et al., 2015). In accordance with this, decreased adiponectin was noted in adipocytes suggesting the prevalence of hypoxia-induced oxidative stress (OS) as also depicted by increased ADMA. Thus, hypoxia seems to be the cause for the differential expression of adipokines in favouring adipocyte maturation restriction as an adaptive strategy to enable the fish to survive under diverse stress conditions.

Zilberfarb et al. (2001) reported that TNFα has inhibitory effects on adipocyte differentiation and thus it is termed as the anti-adipogenic marker. Studies further reported that TNFα suppress adipose conversion by altering the levels of adipokines and adipogenic transcription factors (Hector et al., 2007; Kurebayashi et al., 2001). Consistently, increase in the expression of TNFα along with elevated leptin and declined adiponectin, PPAR-γ, and C/EBPβ, were observed in fish adipocytes of field hypoxic origin and in adipocytes under *in vitro* hypoxia. Thus the results depicts that hypoxia has the strong efficiency to disturb adipokines, thereby it executes the restriction of adipocyte maturation through modulating adipogenic transcription factors, and it evidences that TNFα by its own or by recruiting leptin deregulate the adipostat mechanism by exhibiting lipolytic effects or inhibiting lipogenic process.

Adipogenic transcription factors such as PPAR-γ and CEBPβ, responsible for the maturation process, are downregulated in adipocytes experiencing hypoxia. Consistent with this, recent reports (Trayhurn, 2013; Park and Park, 2010) demonstrated that inhibition of adipocytes differentiation is the major effect caused by hypoxia involving the lifting up of HIF-1α and its mediated effect on adipogenic transcription factors. Increased expression of HSP70 (reported in Padmini et al., 2016a) also regulates PPAR-γ expression via the proteasomal degradation promoted by CHIP (C-terminus of HSC70-interacting protein) and HSP70 interaction (Kim et al., 2017). Apart from HIF-1α and HSP70, leptin is also believed to be involved in the downregulation of PPAR-γ and CEBP-β (Wang et al., 2012; Schroeder et al., 2007). Coherently, in the present study adipocytes of field hypoxic origin and *in vitro* hypoxia expressed the decrease in the PPAR-γ and CEBP-β. This may imply that the prime role of hypoxia is elicited through HIF-1α via suppressing PPAR-γ and CEBP-β. The current study provides direct insight into the value of oxygen, particularly in adipogenesis, stating that oxygen serves as an important environmental cue to promote or inhibit adipocyte differentiation via many different checkpoints. We elucidated that PPAR-γ and CEBP-β serves as one of the key checkpoints determining the adipocyte differentiation into mature adipocytes.

All processes in adipocytes, including adipocyte differentiation and metabolism, depend on the blood flow to the adipose tissue and any disturbance in the blood perfusion may lead to adipocyte hypoxia (Netzler et al., 2015). NO is a potent vasodilator regulating blood flow and vascular function, and appears to be an important mediator of adipocyte physiology with lipogenic properties by stimulating the expression of adipogenic marker, PPAR-γ (Engeli et al., 2004; Yamada et al., 2015). Any alteration in NO can lead to disturbance in adipogenesis via lipolysis which in turn affects the adipose tissue biology (Engeli et al., 2004). ADMA, the endogenous inhibitor of NO, was found to be enhanced in adipocytes of field hypoxic origin and in adipocytes subjected to *in vitro* hypoxic condition. Minakuchi et al. (2016) reported that increased ADMA expression inhibits TG accumulation and adipocyte differentiation. The elevated ADMA observed in the present study may account for causing detrimental impression on the adipose vasculature by inhibiting NO, which in turn leads to perturbations in adipogenesis.

SIRT2 is inversely related to the adipocyte differentiation and adipocyte mass (Jing et al., 2007). It is one of seven in the sirtuin family more widely distributed in adipose tissue than other sirtuins. Studies reported that under limited access to food (caloric stress), FOXO1 proteins are activated by deacetylation evoked by SIRT2 leading to inhibition of adipogenesis through its binding to PPAR-γ and subsequent repression on PPAR-γ transcriptional activity (Armoni et al., 2006; Wang et al., 2008). In agreement to this, decreased SIRT2 was observed in fish adipocytes of field hypoxia and in control on *in vitro* hypoxia revealing their impression on adipocyte maturation. Dryden et al. (2003) reported that cells overexpressing SIRT2 have an extended mitotic phase and progression of the cell cycle. Since adipocyte maturation necessitates the cells to exit from the cell cycle, increase in SIRT2 further adds evidence on suppression of adipocyte maturation under hypoxic condition. In contrast, adipocytes from field hypoxic fish exhibit a decrease in SIRT2 on further subjection to hypoxia in the laboratory. As sirtuins are NAD-dependent deacetylases, their downregulation portrays the deficiency of NAD and it may be due to utilisation of NAD under further lipolysis and β oxidation (Michael, 2017) on *in vitro* hypoxia. Our study reveals that expression of adipogenic transcription factors were not held by SIRT2 under prolonged hypoxia. It is represented by differential expression of SIRT2, adipokines and adipogenic transcription factors in adipocytes from field hypoxia and *in vitro* hypoxia. The current study is the first to report the expression of SIRT2 and its relationship with the adipocyte maturation process during the hypoxic condition.

This altogether implies that adipocytes adapt the hypoxic stress mainly via catabolic signature (lipolysis) which in turn exerts the adipocyte maturation restriction in grey mullet surviving Ennore estuary. Hence, adipocyte maturation restriction elicited by adipokines, adipogenic and anti-adipogenic factors serves as the essential adaptive mechanism triggered by adipocyte in fish surviving in the polluted environment. When the adipocytes isolated from control site are subjected to hypoxia in the laboratory, the results observed in these cells are similar to those isolated from the polluted site which experience hypoxia in the field condition due to pollutants. Thus, adipocyte maturation restriction is tightly regulated by SIRT2 which serves as a downstream signalling molecule of adipokines such as TNFα and leptin. Activation of SIRT2 plays a crucial role in driving away the preadipocytes from the maturation process. Thus the current study hypothesises that sirtuin expression during hypoxia may play a significant role in maintaining adipocyte maturation, which in turn serves as an adaptive strategy in fish inhabiting polluted estuary.

## MATERIALS AND METHODS

Care and use of fishes followed relevant comprehensive guidelines of local animal welfare laws, guidelines and policies.

### Study site and study animal sampling

Grey mullet (*n*=20) with an average length of 30-32 cm were collected from Kovalam (control) and Ennore (test) estuaries using baited minnow traps, which were situated on the east coast of India. Contamination of this estuary by heavy metals and the difference in physical, chemical and biological characteristics has already been confirmed by previous studies (Padmini and Vijaya Geetha, 2007a,b; Padmini and Parimala, 2014). Hypoxic grade of Ennore estuary was also previously reported based on DO level in estuarine water (Padmini et al., 2016a). Fish were collected from both estuaries and placed immediately into insulated containers filled with aerated estuarine water at ambient temperature (25-30°C) and salinity (24-29 ppt). Utmost care was taken to minimise the stress to fish during collection and transport. Fish were maintained in the above specified conditions for 4–5 h until the start of the experimental procedure for the isolation of adipocytes. The experiments were divided into three batches with minimum five samples at each time.

### Adipocytes isolation

Adipose tissue was carefully removed from each fish (*n*=20), then it was washed with distilled water separately and the adipocytes were isolated by the method of Rodbell (1964) with some minor modifications (at temperature 18°C). Briefly, adipose tissue was cut into small pieces and incubated in polypropylene tubes with isosmotic Krebs's buffer (pH 7.4, 280 mM) containing collagenase type II (0.3 mg/ml) and 1% BSA without glucose for 60 min in a water bath under gentle shaking at 18°C. The cell suspension was filtered through a 100 µm filter to remove large undigested tissue particles and centrifuged at 700 ***g*** for 10 min. Then the pellet was washed by flotation. Finally, floating cells were carefully removed as it contains mature adipocytes. One part of the isolated adipocytes from each fish (control-normoxic and test-field hypoxic fish) was separately used for following experiments without subjecting to *in vitro* hypoxia. Another part of isolated adipocytes was subjected to *in vitro* hypoxia and the method follows.

### Incubation of adipocytes under hypoxia

A known volume containing 1×10^4^ isolated adipocytes (both control and test) was immediately suspended in Dulbecco's modified Eagle's medium (DMEM) containing 10% FBS, 2 mM L-glutamine, 10 mM HEPES, and 9 mM bicarbonate antibiotics. Condition of hypoxia was carried out by incubating adipocytes in 1% O_2_; 94% N_2_; 5% CO_2_ for 1 h in the Trigas Forma water jacketed CO_2_/O_2_ incubator (Model: 3131, Thermo Fisher Scientific, USA) with the temperature maintenance of 18°C (Soitamo et al., 2001; Padmini et al., 2016a,b). Following hypoxic incubation, cell viability was assessed by the following method to determine the effect of hypoxia, then it was utilised for the further comparative studies on biochemical analysis.

[Note: As incubation of adipocytes under 1 h hypoxia with 1% O_2_; 94% N_2_; 5% CO_2_ at 18°C mimic field hypoxia based on cell viability, 1 h hypoxia was adopted for studying the adipocytes response to field hypoxia. Other signalling proteins such as HIF1α and HSP70 also depicted the similar pattern of expression in field and control adipocytes on *in vitro* hypoxia. Hypoxia for 2 h and 3 h portrayed their detrimental effect on cell viability. 5% CO_2_ was also standardised for hypoxic studies (Padmini and Tharani, 2015; Padmini et al., 2016a,b).]

### Cell viability assay

Viability of adipocytes (both field condition and incubated under hypoxia) was determined by Trypan Blue staining (Strober, 2001). This dye exclusion test was used to determine the number of viable cells present in a cell suspension and was based on the principle that live cells possess intact cell membrane that exclude dyes such as Trypan Blue, whereas dead cells do not exclude dyes. In brief, suspension cells are harvested by centrifugation. An equal volume of 0.4% (w/v) Trypan Blue is added to a cell suspension at a concentration of approximately 1×10^6^ per ml. The cells were then incubated for 3 min and loaded into a hemacytometer. Nonviable, deep blue cells as well as viable, clear cells are counted in three separate fields using brightfield optics. The viability percentage was calculated by dividing the number of viable cells by the number of total cells and multiplying it by 100.

### Histochemistry analysis

For the cytopathological examination, adipose tissue dissected from control and test fish samples and both incubated under *in vitro* hypoxia for 1 h were mixed with clear 3% agar solution and allowed to solidify by incubating it in freezer. The solidified samples were fixed in 10% phosphate buffered formaldehyde, embedded in paraffin, sectioned at 7 µm, stained with hematoxylin-eosin and periodic acid schiff (PAS) stains and examined by light microscope.

### Transmission electron microscopy

Adipocytes of field hypoxic origin and *in vitro* hypoxic condition were fixed for transmission electron microscopy with 3% glutaraldehyde in 0.1 M sodium cacodylate buffer, pH 7.4, at 4°C. The cells remained in this primary fixative for 2 days. After primary fixation, the cells were washed in sodium cacodylate buffer and postfixed in 1% osmium tetroxide for 1 h at room temperature in sodium cacodylate buffer. Fixation was followed by dehydration of cells by ascending series of graded alcohol (10% to 100%) and propylene oxide. The cells were infiltered, embedded in siliconized rubber mould with epoxy resin and incubated at 60°C for 48 h, for the preparation of blocks for sectioning. Thick sections (1 µm) were cut through ultra microtome (Leica ultracut UCT) with a glass knife and stained with Toluidine Blue dye. The sections were then examined by light microscopy to select areas for fine structural study and photomicrography. Ultrathin sections (below 100 nm) were cut through ultramicrotome (Leica) with diamond knife (Diatome). The ultrathin sections were taken on copper grid and stained with 2% alcoholic uranyl acetate and Reynold's lead citrate solution. The samples were viewed at 80 kV with an electron microscope (201C; Philips Electronic Instruments, Inc., Mahwah, NJ, USA).

### Estimation of triglycerides

Triglyceride was estimated by using commercially available kit based on GPO (glycerol phosphate oxidase) method (Padmini et al., 2016).

### Assay of GPDH

GPDH activity was measured by following Sottile and Seuwen (2001) with slight modifications. Cells were washed with PBS and the assay mixture was added to the tube (0.1 M triethanolamine, 2.5 mM EDTA, 0.1 mM b-mercaptoethanol, and 334 mM NADH, pH 7.7) and tubes were incubated for 10 min at 30°C. The reaction was started by adding 4 mM dihydroxyacetone phosphate. GPDH activity was measured spectrophotometrically at 340 nm. GPDH served as the standard for the assay and results were expressed as mU/mg protein (1 U=1 mmol NADH/min).

### Quantification of adipokines, adipogenic markers and anti-adipogenic markers using ELISA kit

Adipokines such as leptin, adiponectin, TNFα, adipogenic markers such as PPAR-γ, C/EBP-β, anti-adipogenic markers such as ADMA and SIRT2 were quantified using ELISA kits (11-LEPHU-E01, Alpco Diagnostics, USA; 47-ADPHUT-E01, Alpco Diagnostics, USA; K0331131, Koma Biotech, Korea; MBS005886, MyBiosource, USA; MBS006925, MyBioSource, USA; MBS264847, MyBioSource, USA; MBS076909, MyBioSource, USA) according to the manufacturer's instructions.

### Statistical analysis

Data were analysed using statistical software package version 7.0 [IBM SPSS Statistics Subscription Trial (pack)]. One-way analysis of variance (ANOVA) was used to ascertain the significance of variations between control and test fish adipocytes with hypoxia and without hypoxia. Differences were considered significant at *P*<0.05, *P*<0.01 and *P*<0.001.
